# Singlet oxygen-based electrosensing by molecular photosensitizers

**DOI:** 10.1038/ncomms16108

**Published:** 2017-07-14

**Authors:** Stanislav Trashin, Vanoushe Rahemi, Karpagavalli Ramji, Liselotte Neven, Sergiu M. Gorun, Karolien De Wael

**Affiliations:** 1 AXES Research Group, Department of Chemistry, University of Antwerp, Groenenborgerlaan 171, 2010 Antwerp, Belgium; 2 Department of Chemistry and Biochemistry and the Center for Functional Materials, Seton Hall University, 07079 New Jersey, USA

## Abstract

Enzyme-based electrochemical biosensors are an inspiration for the development of (bio)analytical techniques. However, the instability and reproducibility of the reactivity of enzymes, combined with the need for chemical reagents for sensing remain challenges for the construction of useful devices. Here we present a sensing strategy inspired by the advantages of enzymes and photoelectrochemical sensing, namely the integration of aerobic photocatalysis and electrochemical analysis. The photosensitizer, a bioinspired perfluorinated Zn phthalocyanine, generates singlet-oxygen from air under visible light illumination and oxidizes analytes, yielding electrochemically-detectable products while resisting the oxidizing species it produces. Compared with enzymatic detection methods, the proposed strategy uses air instead of internally added reactive reagents, features intrinsic baseline correction via on/off light switching and shows C-F bonds-type enhanced stability. It also affords selectivity imparted by the catalytic process and nano-level detection, such as 20 nM amoxicillin in μl sample volumes.

## Abstract (web summary)

Application of enzyme-based sensors is usually affected by costs, enzyme stability and immobilization and use of additional chemicals. Here, the authors show a cost-effective and robust photoelectrochemical detection system that can mimic enzymatic sensors using only air and light.

The use of enzymes for chemical analysis is well documented. Horse radish peroxidase (HRP), a typical example, has been employed either as a selective catalyst to transform an analyte into an easily detectable product^1^,^2^ or as an enzymatic label in immunosorbent assays and related techniques^3^,^4^,^5^. The advantage of enzymes is their catalytic signal amplification, which translates into high sensitivity and low limits of detection (LOD). Enzyme-based reagents, in combination with a cost efficient and straightforward electrochemical detection method could lead to portable, selective and sensitive sensors^6^,^7^ not unlike the widely used personal glucose meters^8^.

Several research groups have advanced over last three decades the electrochemical detection of phenols at HRP modified electrodes^9^,^10^,^11^ as well as the use of HRP and alkaline phosphatase (ALP) as enzyme labels in electrochemical immunoassays^12^,^13^,^14^. However, despite these efforts and commercial interests, progress in commercialization of electrochemical biosensors remains slow^15^,^16^,^17^. The reasons include the complexity of fabrication of sensing elements, the thermal and chemical instability of enzymes during fabrication, sterilization and storage^18^, the reproducibility of enzyme activity and immobilization procedures, as well as challenges related to field-use of reagents, for example unstable hydrogen peroxide (H_2_O_2_). Thus, sensitive yet robust, renewable reagents and simple, reliable detection approaches are needed.

The elegant idea of using light to activate the chemical conversion of an analyte has been recently introduced in the field of electrochemical analysis^19^,^20^. Chronoamperometry using disposable, screen-printed electrodes (SPE) yields a detection platform similar to that of glucose meters. In the case of so-called photo-electrochemical sensors an analytical signal (photocurrent) is triggered only by light and thus it can be cleanly distinguished from background by simply switching the light off. Advantageously, relatively stable reagents could be photoactivated to start a measurement. For example, an illuminated semiconductor can become a strong electron acceptor, capable to oxidize an organic compound and exchange electrons with an electrode^21^.

Here, we describe a detection paradigm based on the reliability of photoelectrochemical detection and the principles of enzyme detection, that is, catalytic signal amplification, but translated into a bioinspired catalyst that needs only air and light to function. The active, bioinspired enzyme replacement is a Type II molecular photosensitizer which, under visible light illumination generates reactive singlet delta oxygen, ^1^O_2_, which, in turn triggers the appearance of a photocurrent due to its, or its daughter species (Reactive Oxygen Species, ROS) reactions with analytes such as amoxicillin. In addition, we show that photosensitizers such as Pheophorbide A coupled to biomolecules allow a singlet oxygen based DNA–DNA recognition with on-demand reactivity controlled by switching the light on/off.

## Results

### Enzymatic and photosensitizer electrode mechanisms

**Figure 1:**
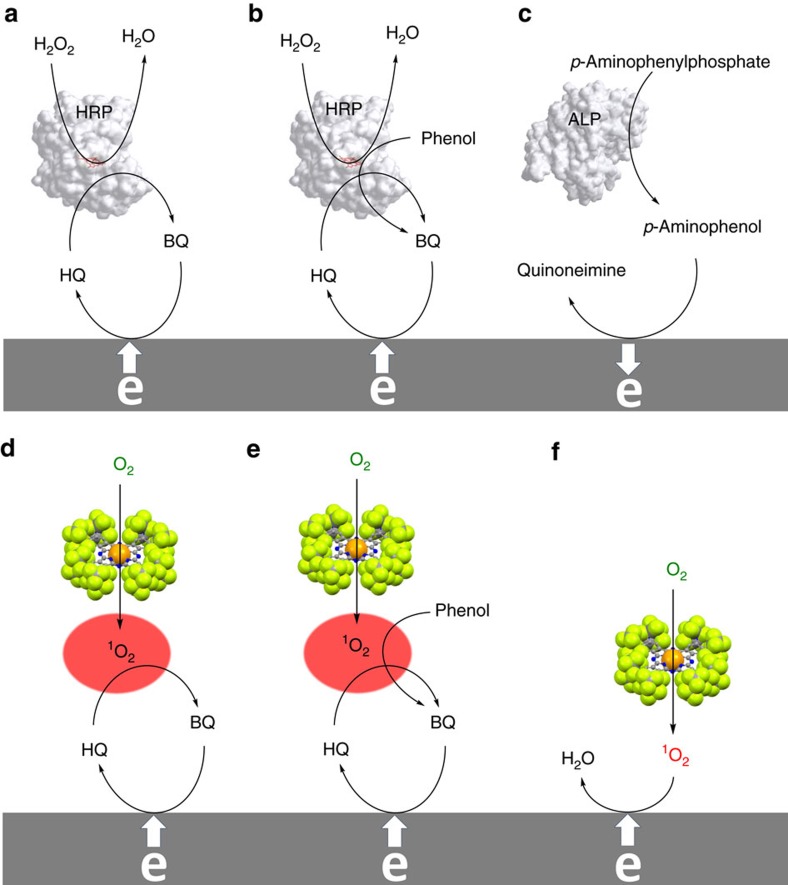
Electrode mechanisms. The white arrows indicate the flow of electrons to/from electrodes. Schematic illustrations of enzymatic reactions mechanisms (**a**–**c**). Mechanisms (**a**) and (**c**): the electrochemical detection of enzyme-labelled reagents. Mechanism (**b**): the detection of phenolic compounds. Schematic illustrations of photosensitized reactions mechanisms (**d**–**f**). Mechanisms (**d**,**e**): photosensitized analogous mechanisms of (**a**,**b**), respectively. Mechanism (**f**): schematic representation of the generation of singlet-oxygen, its reduction and the final production of water as the result of activity of the photocatalyst. ALP, alkaline phosphatase; BQ, benzoquinone; HQ, hydroquinone; HRP, horseradish peroxidase.

Figure 1 illustrates the conceptual parallel between the enzymatic and photosensitizer electrode mechanisms. Specifically, an oxidant, viz H_2_O_2_ or O_2_ interacts with a catalyst, viz HRP or a photosensitizer yielding highly reactive intermediates, namely a ferryl heme or ^1^O_2_, respectively. These intermediates, in turn, rapidly oxidize a substrate, for example hydroquinone (HQ) or another appropriate electron donor in the case of HRP^22^. The HRP oxidant is the iron-oxo core, a classical electron plus proton abstractor, to yield from HQ water and the oxidized product, benzoquinone (BQ). The BQ is electrochemically reduced at a polarized electrode to regenerate HQ thus completing a catalytic redox cycle (Fig. 1a,b). Alternatively, a bio- or photo-catalyst could irreversibly convert an initially redox-inactive reagent into a redox-detectable product, quantifiable electrochemically (Fig. 1c). A lower sensitivity is noted for pathway 1c relative to the redox cycling schemes 1a and 1b, but this scheme's simplicity and low background renders it popular for immunoassays. In contrast, the redox schemes dependency on electron donors, for example phenols makes them useful for their quantification^9^,^10^,^11^. The proposed enzymatic-like approach relies on catalysed photo-transformation of dissolved, aerobic ^3^O_2_ in the reactive, short-lived ^1^O_2_. Advantageously, this approach needs neither H_2_O_2_, unlike HRP-based techniques (Fig. 1a,b) nor *p*-aminophenyl phosphate, unlike ALP-based ones (Fig. 1c).

### Structural and spectroscopic features of the photosensitizer

**Figure 2:**
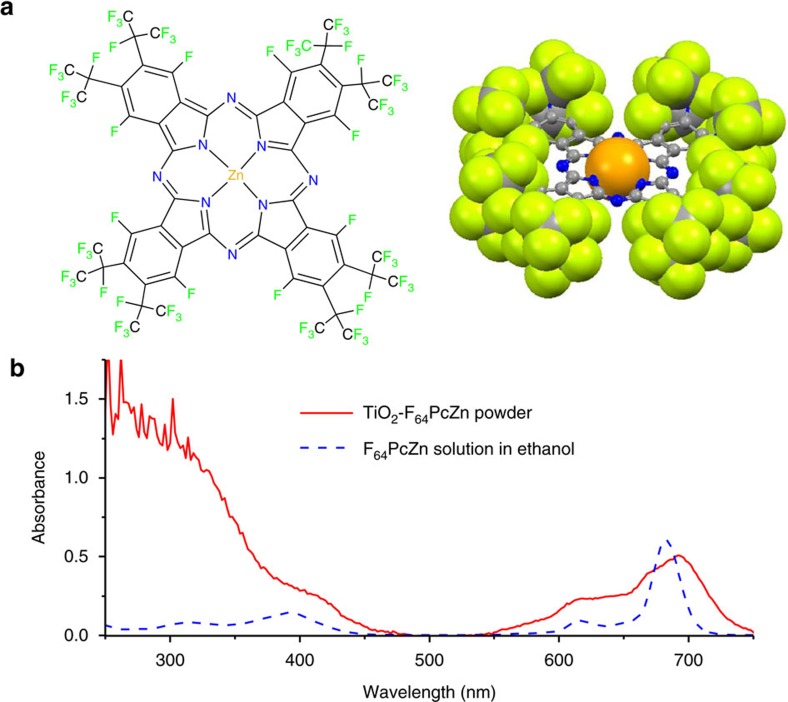
Structural and optical features of F_64_PcZn. (**a**) Structural formula and X-ray structure depiction. Color code and representation: C, gray, ball-and-stick; N, blue, ball-and-stick; F, green, space-filling model, van der Waals radii; Zn, orange, space-filling model, van der Waals radii. (**b**) Diffuse reflectance ultraviolet–visible spectrum of TiO_2_-F_64_PcZn dry powder in comparison with the ultraviolet–visible spectrum of 10 μg ml^−1^ F_64_PcZn in ethanol.

The proposed, chemically robust perfluorinated phthalocyanine complex F_64_PcZn (Fig. 2a) produces ^1^O_2_ in a relatively high quantum yield^23^,^24^,^25^. Unlike the protio phthalocyanine, H_16_PcZn, F_64_PcZn is stable in the presence of ^1^O_2_ and daughter, oxygen-centered species and other radicals^26^,^27^. The bulky *i*-C_3_F_7_ groups of F_64_PcZn effectively protect this molecule from aggregation and facilitate its dissolution in organic solvents such as ethanol. Site isolation is an important feature since aggregation, exhibited by the fluorinated F_16_PcZn diminishes the ^1^O_2_ yield due to the shortening of the excited states lifetimes and/or inefficient intersystem crossing. In addition, the aromatic fluorines of F_16_PcZn are subject to nucleophilic attack, including from some ROS species thus rendering this molecule less viable for ^1^O_2_ photocatalysis^28^. In contrast, attempts to detect nucleophilic substitutions at the 8 β C-F positions of F_64_PcZn have not succeeded thus far, due at least in part to the steric hindrance afforded by the eight adjacent α bulky *i*-C_3_F_7_ groups. This observation further recommends the proposed F_64_PcZn for sustained ^1^O_2_ production.

In summary, the F_64_Pc scaffold affords chemically and thermally stable yet photoreactive complexes^26^,^27^ features that led to its choice. Furthermore, the need for solid-state catalysts using the F_64_Pc scaffold led to the exploration of its support on materials, including TiO_2_ (ref. 29). F_64_PcZn was deposited in 0.5 to 3 wt% on TiO_2_, a carrier matrix with a 21 nm primary particle size and 35–65 m^2^ g^−1^ surface area. The diffuse reflectance ultraviolet–visible spectrum of the hybrid material, TiO_2_-F_64_PcZn, exhibited the characteristic Q-band^30^,^31^ of phthalocyanines in the 600–700 nm range and absorbance below 400 nm, attributed to a combination of Soret bands and the intrinsic absorbance of TiO_2_ (Fig. 2). The Q-band is relatively broad, but centered at the same position as that of F_64_PcZn in solution. The 655 nm wavelength of a common diode laser pointer essentially matches the Q band of TiO_2_-F_64_PcZn, a material that was shown to produce ^1^O_2_ using visible and filtered red light^29^ Thus, a photoelectrochemical response of F_64_PcZn-TiO_2_ deposited on screen printed electrodes, abbreviated SPE|TiO_2_-F_64_PcZn should be absent in the absence of red-light exposure, but could be measurable under illumination. Importantly, dark measurements automatically reveal the actual baseline position, leading to an efficient and straightforward way for baseline correction even in the presence of analytes.

### Oxygen- and electron-based photocatalysis

**Figure 3:**
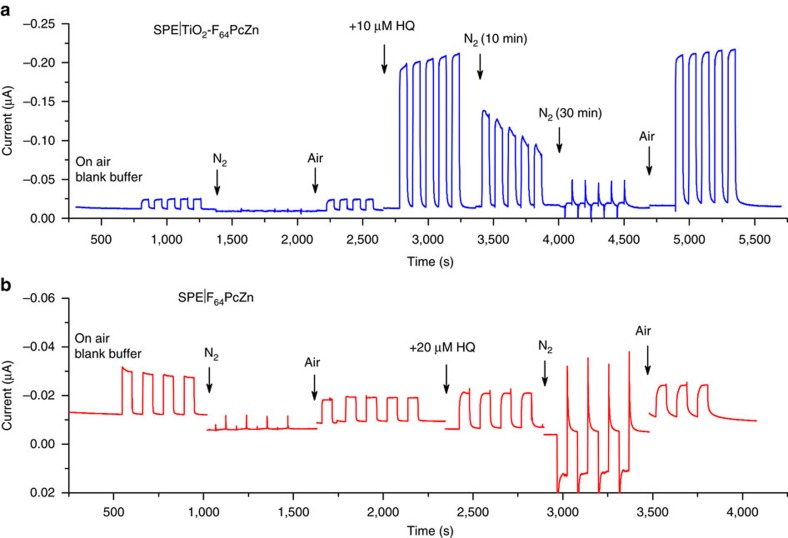
The effect of O_2_ on the photocurrent. The amperometric response was recorded for SPE|TiO_2_-F_64_PcZn (**a**) and directly adsorbed F_64_PcZn, SPE|F_64_PcZn (**b**). The measurements were performed under 655 nm red laser illumination, in the blank buffer (0.1 M KCl, 0.02 M KH_2_PO_4_, pH=7) and in the presence of 10 μM HQ. Amperometry voltage: −0.1 V.

The essential role of O_2_ for substrate detection using the photodynamic effect observed for F_64_PcZn in solution^24^,^25^ and *ex vivo*^23^,^32^, was confirmed by comparing photocurrents obtained in an air-saturated buffer with those obtained under an inert N_2_ atmosphere (Fig. 3). The photocurrents were completely suppressed on N_2_ purges, whether HQ was present or not, but recovered completely on admission of air, consistent with a Type II mechanism and the high ^1^O_2_ quantum yield (*Φ*_Δ_∼0.6 in acetone, methanol) previously reported for F_64_PcZn^23^,^24^,^25^,^32^. Intriguingly, for SPE|F_64_PcZn the anaerobic photocurrent (under N_2_) exhibits a sign reversal in the presence of HQ, suggesting a direct electron transfer from HQ to F_64_PcZn and, eventually to the electrode, in agreement with a recent report on the direct photoreduction of F_64_PcZn by an electron donor in strictly oxygen-free conditions^30^. In other words the photo-oxidation of HQ, which is atom-based (^1^O_2_) in the presence of air, shifts to electron based in O_2_ absence: the SPE accepts electrons from the reduced, anionic F_64_PcZn thereby regenerating continuously the original SPE|F_64_PcZn. This mechanism is specific only to SPE|F_64_PcZn since the lack of electronic connection between the SPE surface and F_64_PcZn in SPE|TiO_2_-F_64_PcZn precludes the appearance of a photocurrent in the absence of O_2_.

The photo-anaerobic signal observed for SPE|F_64_PcZn, given the extreme electron deficiency of the perfluorinated F_64_Pc scaffold might also be considered the basis for a versatile anaerobic sensor application, but this research direction is not pursued in this work.

### Dependency on electrodes and applied potentials

**Figure 4:**
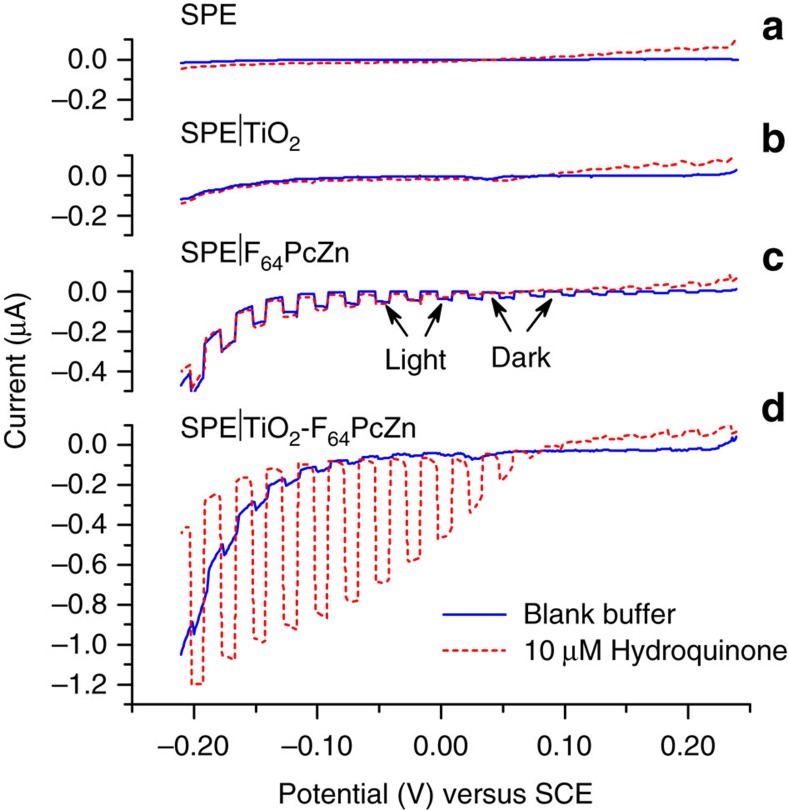
The effect of the electrode modification and the applied potential. Linear sweep voltammetry (LSV) traces recorded for the bare electrode, SPE (**a**); the electrodes modified by TiO_2_, i.e. SPE|TiO_2_, (**b**); SPE|F_64_PcZn, (**c**); and SPE|TiO_2_-F_64_PcZn, (**d**) in the absence (solid, black trace) and presence of 10 μM HQ (dashed, red trace). Scans range from 0.24 to −0.21 V with a scan rate of 0.25 mV s^−1^.

Variations in photocurrents function of the applied potential were investigated by linear sweep voltammetry under light-chopped illumination. The SPEs were subjected to alternating illumination and dark periods of 50 s each, with and without the photosensitizer (Fig. 4).

The bare SPE and the SPE|TiO_2_ showed no photocurrent response either in the absence or presence of HQ (Fig. 4a,b). In contrast, a photocurrent response was observed for SPE|F_64_PcZn and SPE|TiO_2_-F_64_PcZn (Fig. 4c,d) due to the formation of ^1^O_2_ and its subsequent reduction at the electrode surface (Fig. 1f). Higher values of the photocurrent are noted in the presence of HQ due to its redox cycling (Fig. 1d). Noteworthy, at similar F_64_PcZn loadings and in the absence of HQ the response of SPE|F_64_PcZn is higher relative to that of SPE|TiO_2_-F_64_PcZn. Moreover, when 10 μM HQ is added, the SPE|TiO_2_-F_64_PcZn photocurrent increases 20-fold, while the response of SPE|F_64_PcZn is unchanged. More detailed amperometric measurements at higher HQ concentrations revealed that SPE|F_64_PcZn is 50-fold less sensitive to HQ relative to SPE|TiO_2_-F_64_PcZn (Supplementary Fig. 1). These observations could be rationalized considering that ^1^O_2_, the photocurrent trigger has a limited lifetime in water, namely 3.5 μs (ref. 33). Taking into account the 2 × 10^−5^ cm^2^ s^−1^ diffusion coefficient of O_2_ in water ^1^O_2_ can diffuse only about 200 nm, commensurate with the thin F_64_PcZn layer of SPE|F_64_PcZn. In contrast, since the TiO_2_-F_64_PcZn layer of SPE|TiO_2_-F_64_PcZn is obviously more voluminous, a portion of the F_64_PcZn is outside the ^1^O_2_ diffusion radius and thus the ^1^O_2_ it produces decays before reaching the electrode, leading to a lower value of the photocurrent. However, once HQ is present the more stable but redox active BQ occupies a much larger diffusion volume and thus all F_64_PcZn of SPE|TiO_2_-F_64_PcZn (same loading as SPE|F_64_PcZn) contributes now to the photocurrent. The significantly higher activity of SPE|TiO_2_-F_64_PcZn versus SPE|F_64_PcZn is due to the highly dispersed state of F_64_PcZn supported on nano-size TiO_2_, as opposed to it being present as a thin film.

The expected photoinactivity of semiconducting TiO_2_ under low-energy, 655 nm illumination was verified by testing the similar SPE|SiO_2_-F_64_PcZn electrode prepared from insulating SBA-15 SiO_2_. The test device exhibited roughly the same activity as SPE|TiO_2_-F_64_PcZn, thus verifying TiO_2_ exclusive role as a supporting matrix^29^ (Supplementary Fig. 2). Coatings of SiO_2_-F_64_PcZn, however are non-uniform and mechanically unstable and thus have not been used further.

Photocurrents in the presence and absence HQ depended quasi-linearly on the photosensitizer loading for both SPE|TiO_2_-F_64_PcZn and SPE|F_64_PcZn (Supplementary Fig. 3), as expected for catalytic processes. A higher linearity is obtained for the matrix supported F_64_PcZn electrodes, consistent with a load-independent reactivity-type imparted by a non-aggregating, single-site catalyst condensed as thin films or supported on oxidic materials. This observation validates the anticipated, beneficial role of steric hindrance in insuring maximum photophysical activity. The photocurrent plateau observed for SPE|F_64_PcZn suggests that thicker F_64_PcZn films provide diminishing electron transfer ability, again consistent with steric site-isolation and lack of intermolecular electron coupling, as additionally evidenced by the EPR of magnetically isolated, isostructural F_64_PcCu^34^.

### Photocurrents dependency on light sources

Amperometry measurements were conducted using different 665 nm laser power settings (Supplementary Figs 4 and 5). The photocurrent increase was steep up to about 20 mW, but tended to diminish when the power exceeded 50 mW. Standard LED lamps emitting 2,100 and 3,000 millicandelas at 660 nm provided a response equivalent to laser powers of 1.2 and 1.7 mW or 15.6 and 22.4% of the sensitivity at 30 mW, respectively.

### Detection of phenolic analytes

HRP was reported to be a promising reagent for the detection of phenols through their oxidation to redox active derivatives^9^,^10^,^11^,^35^,^36^. A Type II photosensitizer is now used for the same purpose, therefore a detection mechanism is proposed (Fig. 1b,e). This proposal is consistent with the reported pathway for photocatalytic degradation of phenol^37^,^38^ and phenolic derivatives^39^,^40^, including pollutants and pharmaceuticals^41^,^42^.

**Figure 5:**
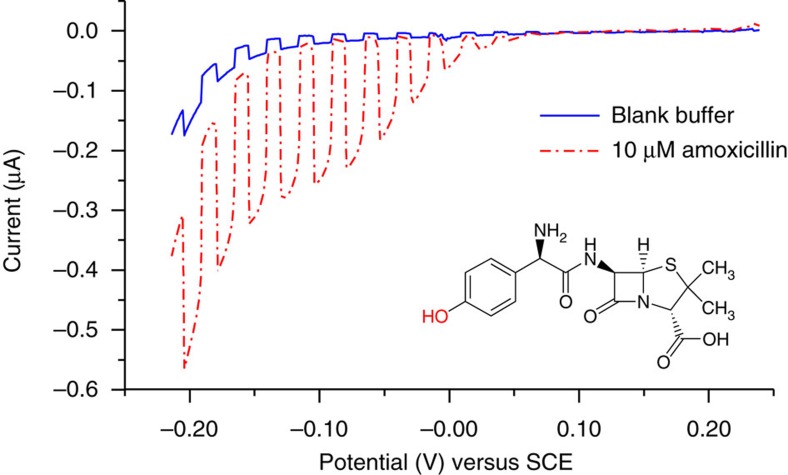
Effect of the electrode potential on amoxicillin detection. Linear sweep voltammetry (LSV) traces recorded for TiO_2_-F_64_PcZn electrode in the absence and presence of 10 μM amoxicillin. Scans range from 0.24 to −0.21 V with a scan rate of 0.25 mV s^−1^.

Amoxicillin, a β-lactam antibiotic containing a phenolic moiety was used to test the efficiency of the proposed detection strategy. Similar to HQ, amoxicillin triggers a potential-dependent rise of the photocurrent (Fig. 5), which remains constant beyond a potential of around −0.1 V versus SCE. By analogy with HQ, a ^1^O_2_-mediated photo-oxidation of amoxicillin yielding a redox active product may occur, followed by the reduction of this product at the electrode. This mechanistic proposal is based on the reported reaction of ^1^O_2_ with the 4-hydroxyphenyl moiety of amoxicillin via a [2+2] cycloaddition which yields an unstable 1,4-endoperoxide that, in turn decays to the corresponding *p*-peroxyquinol^38^,^43^. A minor product, a 3,4-dihydroxyphenyl derivative can be additionally formed, similar to the oxidation of tyrosine by ^1^O_2_ (ref. 44).

How selective is the proposed detection for phenols? Ampicillin, which lacks the aromatic hydroxyl group of amoxicillin, as well as benzylpenicillin, nafcillin, and 6-aminopenicillanic acid produced no noticeable photocurrents even in concentrations as high as 100 μM (Table 1 and Supplementary Fig. 6). Several phenols, were evaluated next (Supplementary Table 1). Setting hydroquinone to 100, the relative sensitivities are: 4-aminophenol, 113; 2-chlorophenol, 44; bisphenol A, 39; amoxicillin, 32. Surprisingly, only a minor response was observed for 4-methylphenol, 1. Phenol, bisphenol A, 4-nitrophenol and 2-chlorophenol have longer response times, 50–100 s, in comparison to HQ, 10 s, likely due to their slow reactions with ^1^O_2_ (ref. 45). Reaction rates increase with the pH^45^, a change to pH 12 results in both a fast response, 5 s, as well as a fivefold increase in sensitivity, as demonstrated for phenol and bisphenol A (Supplementary Table 1).

In contrast to phenols, ascorbic acid is an example of a compound that can rapidly but irreversibly react with singlet oxygen^46^. The lack of redox cycling limits its photocurrent response. Indeed, the sensitivity of the proposed electrode to HQ is three orders of magnitude higher compared to that for ascorbic acid, as evidenced by the appearance of only a minor photocurrent response even when the concentration of ascorbic acid exceeds 100 μM (Supplementary Table 1). Notably, ascorbic acid does not suppress the response of HQ at a 1:1 molar ratio. The photocurrent intensity is halved only if ascorbic acid is present in 100-fold excess. Applications for the detection of phenols in the presence of ascorbic acid, for example in fruits and beverages are thus possible since the phenols are always present in excess^47^.

**Figure 6:**
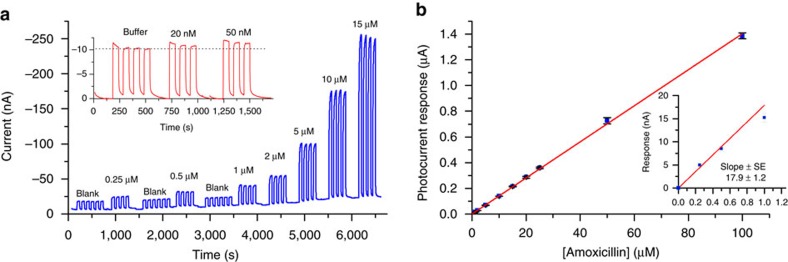
Amperometric detection of amoxicillin. Amperometry measurements traces (**a**) and the calibration curve (**b**) obtained for amoxicillin at −0.1 V in 0.1 M KCl, 0.02 M KH_2_PO_4_, pH=7. Sample volume, 80 μl. Diode laser, 655 nm, 30 mW. Data for the calibration curve are presented as mean (±s.d.) of four consecutive measurements.

Plots of the photocurrent for amoxicillin as a function of its concentration reveal that the sensitivity was 0.14 A M^−1^ cm^−2^ in the low concentration range, corresponding to the limit of detection (LOD) 22 nM, calculated from the 3s.d. value of the background signal (s.d.=0.13 nA, *n*=16) (Fig. 6). The photocurrents for HQ were about three times higher compared to amoxicillin, possibly because of the slower photo-oxidation kinetics for amoxicillin compared to HQ (Supplementary Fig. 7). The sensitivity for HQ was 0.41 A M^−1^ cm^−2^ with a LOD of 12 nM.

Notably, the LOD values for both amoxicillin and HQ were about two orders of magnitude lower compared to a recently reported system using iron phthalocyanine (Type I photosensitizer) designed for dopamine detection^48^, and one-two orders of magnitude lower compared to HRP-modified electrodes used for the detection of phenolic compounds^9^,^10^,^35^,^36^. Similar, favourable results are noted following a comparison with previously published phenolic analytes (Supplementary Table 2), as well as with amoxicillin determinations using different electrochemical methods (Supplementary Table 3).

**Figure 7:**
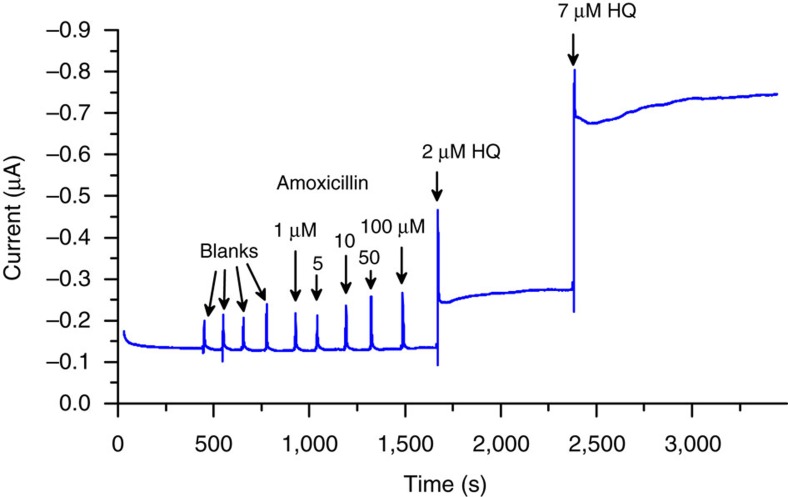
Amperometry measurements at a TiO_2_-HRP modified electrode. The amperometric response curve obtained using a TiO_2_-HRP modified electrode operated under the same conditions as the TiO_2_-F_64_PcZn electrodes. The baseline was stabilized in the measuring buffer (0.1 M KCl, 0.02 M KH_2_PO_4_, pH=7) containing 1 mM H_2_O_2_. The total volume of the measuring solution was 100 μl. Before the introduction a compound into the drop, amoxicillin and HQ were mixed with H_2_O_2_ to keep its concentration unchanged during the measurements. The numbers denote the final concentration of amoxicillin or HQ. Blanks contained only H_2_O_2_.

A direct comparison between F_64_PcZn and HRP modified electrodes provides more insights into the proposed method. To our knowledge, the detection of amoxicillin using an enzymatic biosensor has not been reported. Thus, a SPE|TiO_2_-HRP electrode, analogous with SPE|TiO_2_-F_64_PcZn was constructed. The HRP-based electrode detected HQ with sensitivity similar to that of TiO_2_-F_64_PcZn (Fig. 7), but it did not detect amoxicillin, substantiating the advantages of the catalytic, ^1^O_2_-mediated detection strategy.

**Figure 8:**
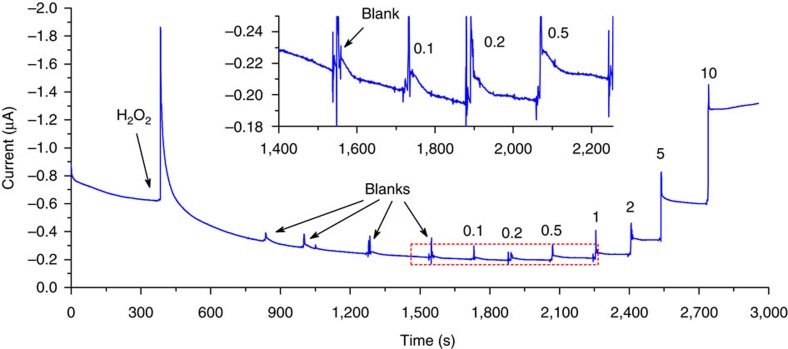
Amperometric detection of HQ at a TiO_2_-HRP-modified electrode. The amperometric response curve obtained using a TiO_2_-HRP-modified electrode, from the start of the polarization of the modified electrode. The numbers denote the final concentration of HQ. Blanks contained only H_2_O_2._ Measuring conditions were the same as in Fig. 7.

The favourable sensitivity reported above is dependent on the signal-to-noise ratio of the detection method. The photocurrent of the SPE|TiO_2_-HRP was observed to fluctuate on the addition of H_2_O_2_ when low concentrations of HQ were measured (Fig. 8), consistent with known H_2_O_2_ problems^49^. Mitigation attempts by disconnecting and reconnecting the electrode exacerbated the baseline instability. A second remedy was tried by maintaining the reaction volume strictly constant. Hence, HQ was introduced by removing 20 μl from the total volume (100 μl) and returning the same volume of HQ solution also containing H_2_O_2_. Thus, measurements were performed continuously without switching off the cell or affecting noticeably the concentration of H_2_O_2_. The addition of 0.1 μM HQ after 20 min of the baseline stabilization elicited no clear response (Fig. 8). The lowest concentration detected was 0.2 μM although the sensitivity of the electrode, 0.39 A M^−1^ cm^−2^ was similar to that of SPE|TiO_2_-F_64_PcZn. The extrapolation of the baseline beyond the time the sample is introduced, however, is problematic rendering the LOD values uncertain.

In this respect, the strategy we suggest presents a distinct LOD advantage since switching off the light reveals the baseline current under the experimental measuring conditions, thereby affording a simple, straightforward correction if necessary. Moreover, while the photoelectrochemical and HRP-based approaches reveal similar selectivity patterns (Supplementary Table 1), the phthalocyanine, unlike HRP is stable at elevated pH values. As mentioned above, a higher pH favours the reaction of phenols with ^1^O_2_ (ref. 45), and might be encountered when measuring analytes in basic wastewaters. In general, enzymes function only within narrow pH ranges, a condition which is less restrictive in the case of chemically robust photosensitizers.

### Photosensitizers as labels of biomolecules

The selectivity based on the specific reactions of ^1^O_2_ could be enhanced by attaching photosensitizers to supports that exhibit binding selectivity. To demonstrate this point, a commercially available oligonucleotide of 15 bases labelled by Pheophorbide A, a chlorophyll-derived, well-known photogenerator of singlet oxygen^50^,^51^ apoporphyrin was used, consistent with its F_64_PcZn-related *ex vivo* reactivity^32^. The oligonucleotide sequence was chosen to be complementary with microRNA-21, an important marker for several types of cancer and cardiovascular diseases^52^. The electrodes were modified by a DNA probe with the complementary sequence, while a non-complementary DNA probe of the same length was used as a blank control.

**Figure 9:**
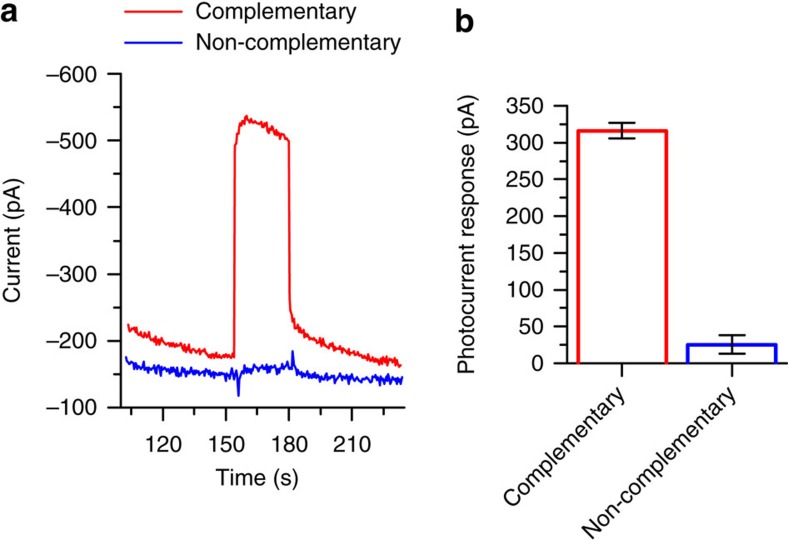
Amperometric detection of the microRNA21 complementary oligonucleotide. Amperometry measurements traces (**a**) and photocurrent responses (**b**) after incubation in 10 nM solution of the oligonucleotide labelled with the Type II molecular photosensitizer. Photocurrents were measured in a three-electrode cell at a constant potential of −0.05 V versus SCE using a diode laser, 655 nm, 30 mW. Data are presented as mean response (±s.d.) of four independent electrodes.

As shown in Fig. 9, the complementary oligonucleotide elicited a clear photocurrent response, but only a very small photocurrent was observed for the non-complementary sequence. The photocurrents, reproducible within a series of four electrodes had an average value of 316 pA (s.d.=11 pA, *n*=4), a number that exceeded ∼12 times the 25 pA (s.d.=13 pA, *n*=4) average photocurrent in blanks. The signals, however, are time-dependent, dropping by 40% after four sequential illuminations of 30 s (Supplementary Fig. 8). The decay was likely due to the oxidative DNA cleavage and, possibly the photodegradation of the photosensitizer. Note that the dark photocurrent remained constant for 40 min after the electrode incubation in the measuring buffer (Supplementary Fig. 8). Thus, the signal decay was likely not due to the dissociation of the labelled oligonucleotide from the electrode surface. Nevertheless, the decay of the photocurrent, which reached its maximum after 5 s of illumination is not critical for the one-time detectability of the complementary oligonucleotide due to this fast response of the sensor.

## Discussion

The use of enzymes for chemical analysis is reaching maturity. Photosensitizers generating ^1^O_2_ have been explored and exploited in the fields of organic synthesis, medical photodynamic therapy and others, but not yet in the field of chemical sensors^53^,^54^. The proposed analogy between the photo-catalytic and enzymatic detection schemes, Fig. 1, might be the basis for new directions of exploration of ^1^O_2_-generating photo-catalytic materials as chemical sensors.

The high reactivity of ^1^O_2_ and its ROS daughters, however, limits the use of photocatalytic materials due to photosensitizers degradation and thus low turnover numbers^54^. This is not surprising considering that C–H bonds containing photosensitizers are being attacked by the ^1^O_2_ and ROS they produce. Moreover, the oxidation of analytes may also generate reactive species, including radicals, an additional source of sensor degradation.

The photosensitizer photo-degradation is mitigated by using a fully fluorinated Zn phthalocyanine complex, F_64_PcZn, an efficient yet stable ^1^O_2_ generator^26^,^27^ in solution and in the solid state. The manufacture of electrodes containing F_64_PcZn revealed that robust, ROS and redox processes inactivation-resistant sensors can be produced. The bulky F_64_Pc organic scaffold, complexed by a closed-shell metal ion like Zn^2+^ precludes deactivating aggregation, exhibits reversible electron addition and resistance to radical, electrophilic and nucleophilic attacks^27^.

Electronic transfers to/from the sensor are thus feasible while the interactions of electrodes with analyte species occur without baseline interference. The latter process is possible since only light switching triggers the appearance of analyte products, the dark photocurrents being obtained from a chemical composition-invariable environment. Compared with enzymatic detection, significantly higher signal-to-noise ratios are generally observed, while selectivity based on the type of chemical oxidation represents an additional bonus.

The proposed strategy compares favourably with HRP-based detection for a series of phenols, including pharmaceuticals bearing the phenol functionality. The analyte chosen as an example, amoxicillin, is the most used antibiotic^55^, but also contaminates hospital trash and urban wastewaters and is therefore a marker in environmental management and pollution control^56^. Amoxicillin and other phenols' selective detection suggests that the direct, ene reactivity of ^1^O_2_ operates, as noted previously for phenol^38^ and dominates the selective sensing process. The shuttle of electrons between a site of ^1^O_2_ production/reactivity and an electrode occurs for both SPE|TiO_2_-F_64_PcZn and SPE|F_64_PcZn electrodes that detect redox-active products, but the nano-dispersed TiO_2_-F_64_PcZn is more effective due to its high surface area and spatial distribution of F_64_PcZn in the large bulk volume of the support. The sensing efficiency can be understood and tuned using classical catalysis principles.

Catalyst robustness, structural modifications favouring single-site isolation, loading degree, light intensity, dark baseline corrections, surface area and particle sizes of supports and so on, could be used to optimize performance. The kinetics of the ^1^O_2_-mediated oxidation of an analyte could be further tuned by modifying the reaction conditions such as temperature and pH, as shown for phenols. The large variation of pH, an important parameter for optimizing sensors' sensitivity and selectivity, is unavailable in the enzymatic detection scheme.

Molecular photosensitizers could also be used as labels for biorecognition/affinity assays even if somewhat unstable, such as Pheophorbide A since the signal decay takes minutes while detection occurs seconds after the catalyst is photoactivated. The life-time of ^1^O_2_ suggests a diffusion distance of about 200 nm and thus the label itself does not need to contact an electrode to produce an analytical response. Application of this detection scheme, however, is rather limited by the single-use type of the sensors due to the response decay in a sequence of repeatable measurements. While electroactive compounds (for example, ferrocene) could still be useful as labels in case of short stranded DNA, they cannot provide an efficient and generic solution for affinity sensors due to the rapid charge transfer rate–distance decay^57^,^58^ that results in poor signals for biomolecules, whose typical size is a few nanometers.

As noted above, the redox cycling afforded by HQ results in remedial signal amplifications due to electrons shuttling between the electrode and the ^1^O_2_ producing photo-catalyst, suggesting opportunities for electrochemical microarrays and washing-free immunosensors^59^.

In conclusion, robust, perfluorinated molecular photosensitizers, resistant chemically yet reactive, have been shown as proof-of-principle efficient enzymes mimics for electrochemical (bio)sensing applications, while favourably enhancing the useful feature of the enzymatic detection mechanism, namely the catalytic formation of an easily detectable product and redox cycling. A Type II photosensitizer generates photocurrents using air oxygen without the need to add any supplementary reagents.

The perfluorination of the sensitizer insures its functional resilience as well as long term stability vis-à-vis the reactive oxygen and other species it may generate. The use of catalytic photosensitizers instead of enzymes for analytical sensing offers several advantages, including: chemical and thermal stability; on-off control of sensing by on-off light switching; facile dark baseline monitoring and adjustments; invariable structural and reactivity properties of photocatalytic, well-defined metal complexes; comparative simplicity and low preparation price; facile chemical modification and functionalization, attachment to biomolecules and surfaces.

The bioinspired molecules, subject to chemical modifications, coupled to biomolecules may allow conjugates to function in the same way as fluorescent dye- and enzyme-labelled (for example, HRP-labelled) reagents, but affording an additional, on-demand reactivity controlled by simply switching the light. An enhanced degree of flexibility in the design of biorecognition elements and the functionalization of sensor surfaces is envisioned. The present example of a bioinspired strategy of replacing an enzyme with synthetic components for analytical purposes could be useful for developing applications ranging from chemistry to biology and environmental monitoring.

## Methods

### Reagents

Perfluorophthalocyanine Zn, F_64_PcZn was synthesized and characterized as described earlier^24^ Briefly, perfluoro-(4,5-bis-isopropyl)phthalonitrile was prepared from perfluorophthalonitrile and perfluoropropene and reacted with Zn acetate. The obtained product was purified chromatographically and recrystalized twice from acetone. The ^19^F NMR and UV–vis spectra were identical with the literature data.

Titanium dioxide, TiO_2_ (Aeroxide P25) and silicon dioxide, SiO_2_ (AEROSIL OX 50) were obtained from Evonik Inorganic Materials (USA).

Before impregnation with F_64_PcZn, SiO_2_ and TiO_2_ were dried at 100 °C for 2 h. After dissolving F_64_PcZn in 10 ml of absolute ethanol, either SiO_2_ or TiO_2_ were added to this solution. The ethanol was evaporated under vacuum and the impregnated materials were dried at 100 °C for 6 h. Loadings of 0.5–3.0% wt F_64_PcZn were confirmed spectrophotometrically by back extracting the phthalocyanine from the impregnated materials with acetone (Soxhlet) until the oxide appeared white and no phthalocyanine was observed via reflectance ultraviolet–visible spectroscopy. TiO_2_ containing 3 wt % F_64_PcZn was used in all experiments, if not mentioned otherwise.

For the impregnation of TiO_2_ with HRP, TiO_2_ was suspended in a solution of 0.125 mM HRP in 10 mM HEPES pH=7 buffer and the mixture was agitated overnight on a rotatory shaker. The suspension was centrifuged and the pellet washed three times with 10 mM HEPES pH=7 buffer and dried at room temperature for 8 h. The resulting powder was refrigerated before use. A loading of 1.06 μmol g^−1^ (4.6 wt %) was calculated from the concentration of HRP in the supernatant collected after adsorption. The supernatants of washing solutions did not contain any noticeable amount of HRP.

Oligonucleotides were obtained from Eurogentec (Belgium). Their structures and purity were confirmed by mass-spectrometry. Sequences of the oligonucleotides were as follows: probe 1 (complementary): 5′-HS-(CH_2_)_6_-tagcttatcagactgatgttga-3′; probe 2 (non-complementary): 5′-HS-(CH_2_)_6_-tagcttatgtgtaccctgtcag-3′; oligonucleotide labelled by Pheophorbide A: 5′-Pheo-tcaacatcagtctga-3′. A detailed description of the procedures used for the modification of electrodes by the probes and detection of the labelled oligonucleotides is provided in the Supplementary Methods.

Amoxicillin of 99.4% purity was obtained from TCI Europe (Belgium), HQ of 99.9% purity was purchased from Acros Organics (Belgium). Other phenolic compounds were 98% purity or better and obtained from different suppliers. L-Ascorbic acid of 99.5% purity was obtained from Sigma-Aldrich (Belgium). Ultrapure water was used for all experiments.

### Equipment

The electrochemical measurements were conducted using a μAutolab III (Metrohm-Autolab BV) instrument. Data for calibration curves were obtained using PalmSens3 (PalmSens BV) instrument. Ultraviolet–visible diffuse reflectance spectra were measured using an Evolution 500 double-beam spectrophotometer equipped with RSA-UC-40 DR-UV integrated sphere (Thermo Electron Corporation) or a Cary 5000 instrument.

A diode laser pointer operating at 655 nm (Roithner Lasertechnik, Austria) was adjusted to 30 mW power using a light power meter. A power supply was programmed to switch on and off the light beam at given time intervals.

### SPE modifications

Screen-printed carbon electrodes (SPE) were purchased from DropSens (Asturias, Spain).

The modification of electrodes by F_64_PcZn to give SPE|F_64_PcZn was performed by depositing a 5 μl drop of 0.3 mg ml^−1^ of F_64_PcZn solution in ethanol on SPEs and letting the solvent evaporate.

The SPE|TiO_2_-F_64_PcZn were manufactured by adding a 5 μl drop of an aqueous suspension containing 10 mg ml^−1^ TiO_2_-F_64_PcZn on the working electrode surface of SPEs and allowing the water to evaporate completely at room temperature.

### Electrochemical measurements

Measurements with SPE were performed in a drop of 80 μl. Measuring buffer consisted of 0.1 M KCl and 20 mM KH_2_PO_4_ (pH=7) dissolved in ultrapure water. A saturated calomel electrode (SCE, Radiometer, Denmark) was used as an external reference electrode whenever necessary. The quasi-reference electrode of SPEs had the potential of +0.04 V versus SCE in the measuring buffer. All potentials in the text are given versus SCE. To study the effect of oxygen, a tightly closed three electrode cell was used with SPE, SCE, and a glassy carbon rod as the working, reference and counter electrodes, respectively. The beam of the diode laser was directed to the working electrode surface through the glass wall of the cell. Some, constant power loss was noted due to the glass wall absorption.

### Data availability

All data is available from the authors on reasonable request.

## Additional information

**How to cite this article:** Trashin, S. *et al*. Singlet oxygen-based electrosensing by molecular photosensitizers. *Nat. Commun.* **8,** 16108 doi: 10.1038/ncomms16108 (2017).

**Publisher’s note:** Springer Nature remains neutral with regard to jurisdictional claims in published maps and institutional affiliations.

## Supplementary Material

Supplementary Information

## Figures and Tables

**Table 1:** Selectivity to the phenolic moiety.

**Compound**	**Photocurrent response**±**s.d. (nA)**
Amoxicillin	1,387±23
Ampicillin	1.2±0.8
Benzylpenicillin	−1.0±0.8
Nafcillin	6.8±3.7
6-aminopenicillanic acid (6-APA)	7.6±2.9

The amplitude of the photocurrent responses to 100 μM concentrations of three different penicillins and the 6-APA penicillin precursor in comparison to amoxicillin. Data are presented as mean (± s.d.) of four consecutive measurements.
