# Supplementary material for: Singlet oxygen-based electrosensing by molecular photosensitizers
Source: Nat Commun. 2017 Jul 14;8:16108. doi: 10.1038/ncomms16108 (PMC5519987; doi:10.1038/ncomms16108)
Supplement: Supplementary Information [file ncomms16108-s1.pdf]

Title of file for HTML: Supplementary Information
Description: Supplementary Figures, Supplementary Tables, Supplementary Methods and Supplementary References

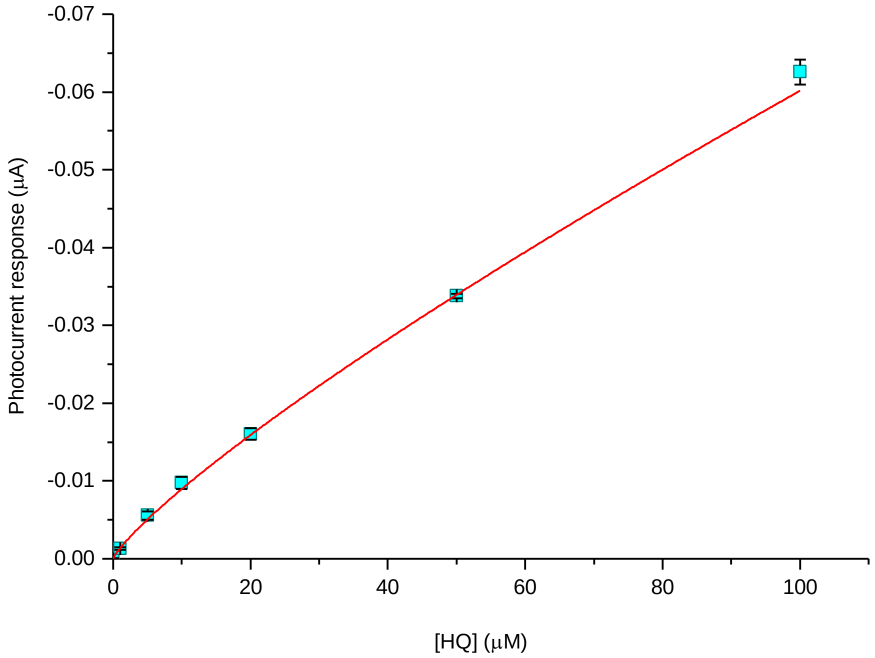

Supplementary Figure 1. Dependence of the photocurrent on the HQ concentration for SPE|F_{64}PcZn. Deposited F_{64}PcZn on SPE: 5 μl of 0.3 mg ml^{-1} solution in ethanol. Potential applied: -0.1 V; background, 0.1 M KCl, 20 mM KH_2PO_4, pH = 7; the photocurrent of the blank buffer was subtracted; Red diode laser, 655 nm, 30 mW. The error bars represent s.d. of four consecutive measurements.

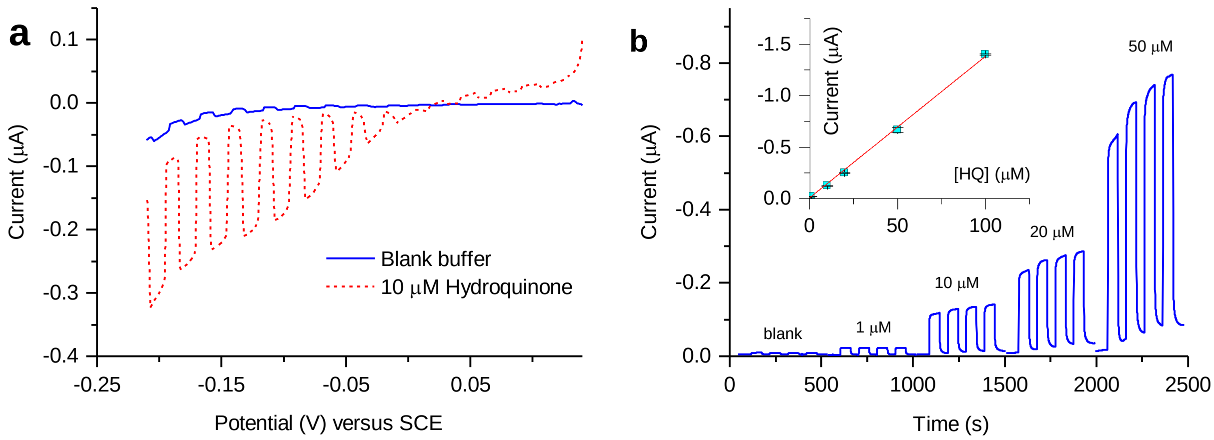

Supplementary Figure 2. Silica as carrier for the photosensitizer. (**a**) Linear sweep voltammetry recorded for SPE|SiO_2-F_{64}PcZn. (**b**) Amperometry measurements at a constant potential of -0.1 V and different concentrations of HQ. Background, 0.1 M KCl, 20 mM KH_2PO_4, pH = 7. Sample volume, 80 µl. Diode laser, 655 nm, 30 mW.

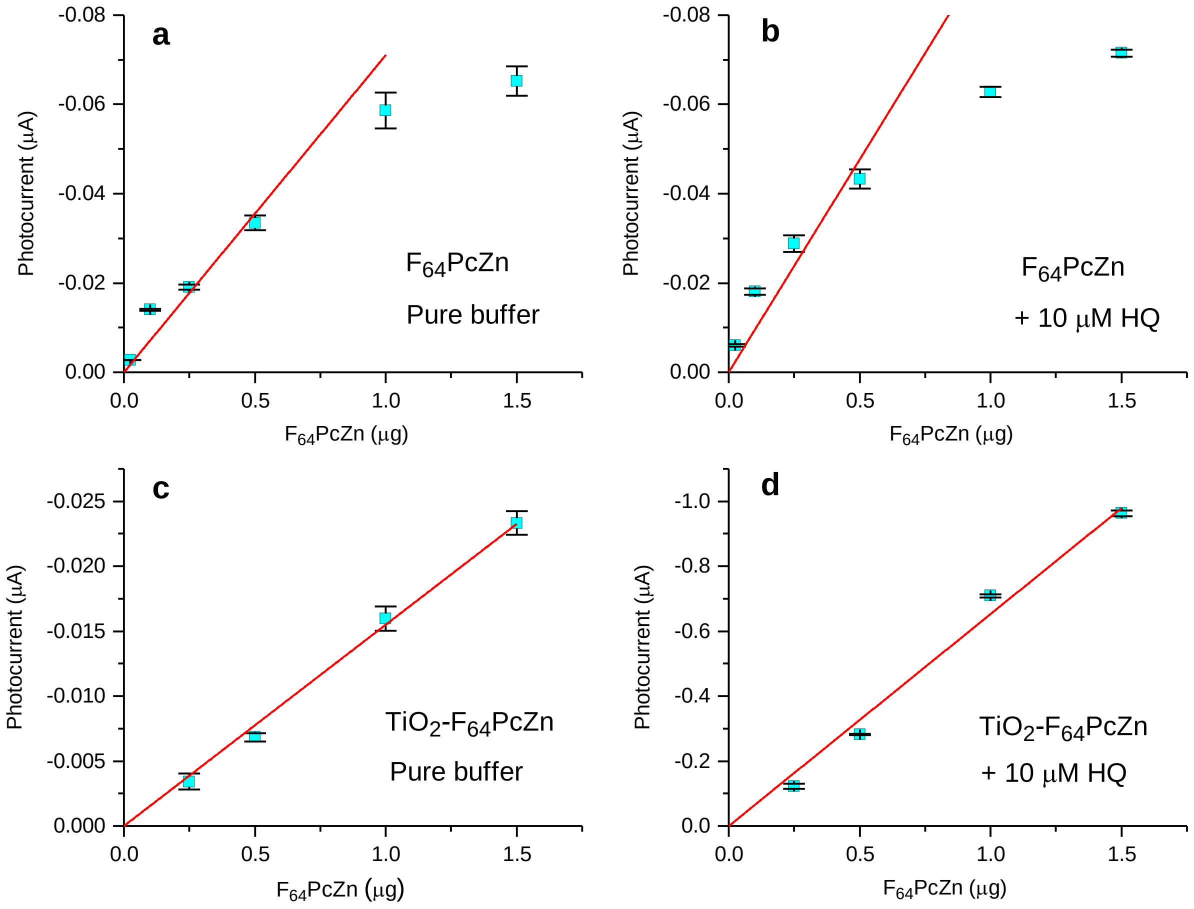

Supplementary Figure 3. Amount of photosensitizer on the electrode. Dependence of the photocurrent response on the amount of $F_{64}PcZn$ directly deposited on the electrode (**a** and **b**) or immobilized onto TiO_2 (**c** and **d**), in the absence (**a** and **c**) and presence (**b** and **d**) of 10 μM HQ. Electrodes with $F_{64}PcZn$ were prepared by drying 5 μl of 0.01–0.3 mg ml^{-1} $F_{64}PcZn$ prepared in water from 3 mg ml^{-1} stock solution in ethanol. Electrodes with TiO_2-$F_{64}PcZn$ were prepared from 10 mg ml^{-1} aqueous suspension of TiO_2 containing 0.5–3 wt% $F_{64}PcZn$. The photocurrent was recorded at a constant potential of -0.1 V vs SCE in 0.1 M KCl, 20 mM KH_2PO_4, pH = 7. Sample volume, 80 μl. Diode laser, 655 nm, 30 mW. The error bars represent s.d. of four consecutive measurements.

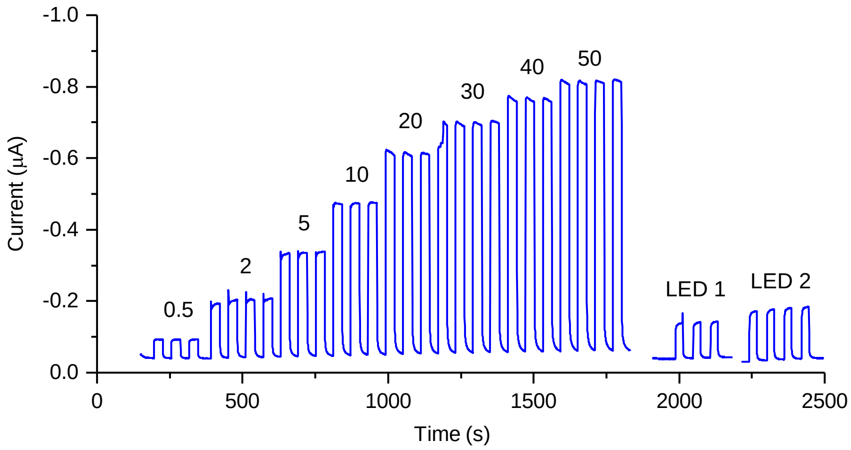

Supplementary Figure 4. Effect of the laser power. Amperometry measurements for TiO_2-$F_{64}PcZn$ recorded in the presence of 10 μM HQ, at an applied potential of −0.1 V. Background electrolyte, 0.1 M KCl, 0.02 M KH_2PO_4, pH = 7. Sample volume, 80 μl. Diode laser, 655 nm. The numbers denote the laser power in mW. For comparison, illuminations with two typical red LED lamps (GaAlAs, 660 nm) are given. LED 1, L-7113SRC-DV, 2100 mcd, of 5 mm in diameter; and LED 2, L-793SRC-E, 3000 mcd, 8 mm in diameter.

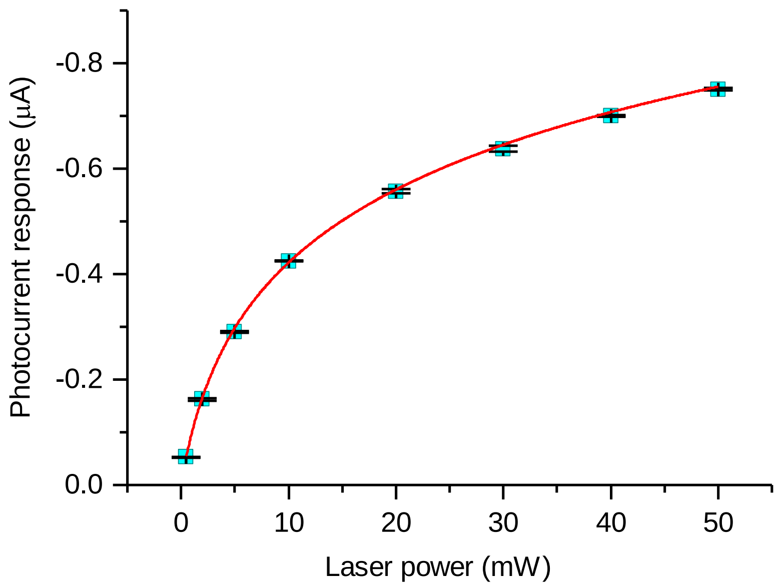

Supplementary Figure 5. **Dependence of the photocurrent response on the laser power.** Conditions are the same as in Supplementary Figure 4. The error bars represent s.d. of three consecutive measurements.

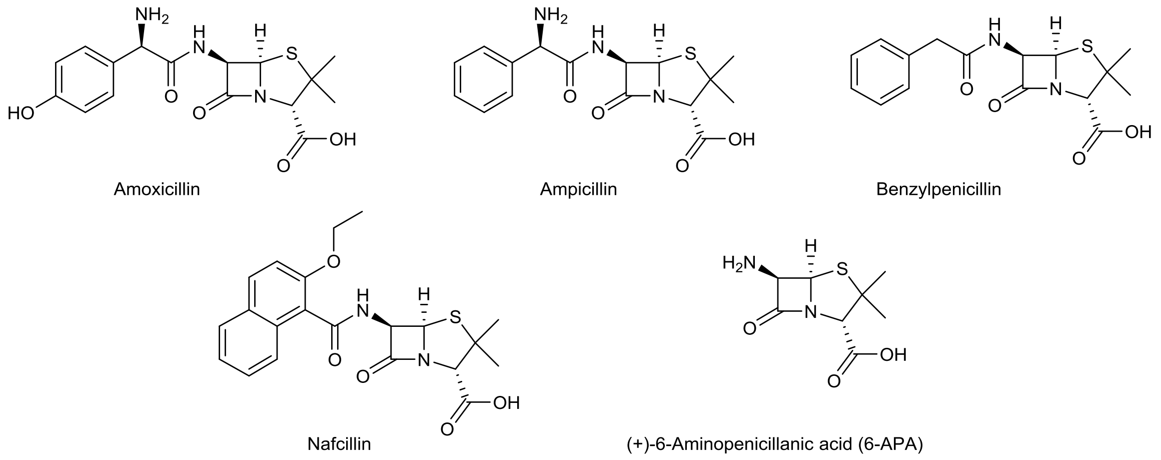

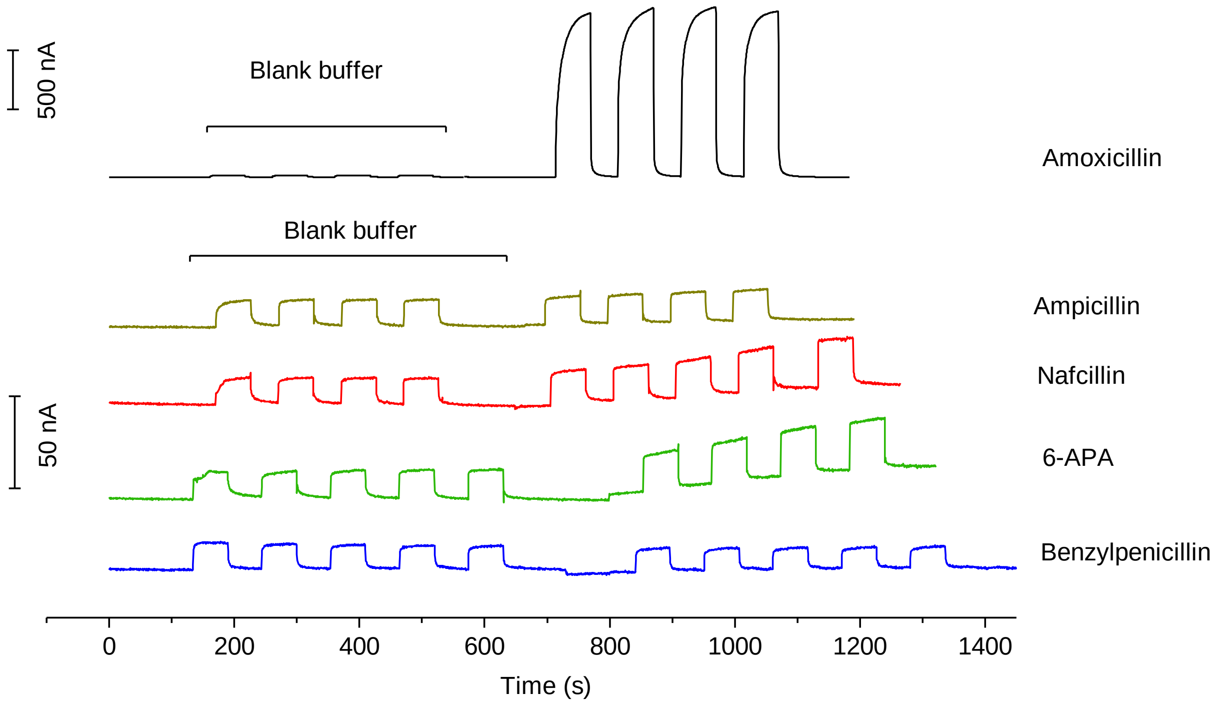

Supplementary Figure 6. Selectivity study. Amperometry measurements at TiO_2-F_{64}PcZn modified SPE at a potential of -0.1 V in pure buffer and in the presence of 100 µM amoxicillin in comparison to three different penicillins and 6-aminopenicillanic acid (6-APA). Potential applied: −0.1 V; background, 20 mM pH = 7 phosphate buffer containing 0.1 M KCl; red diode laser, 655 nm, 30 mW.

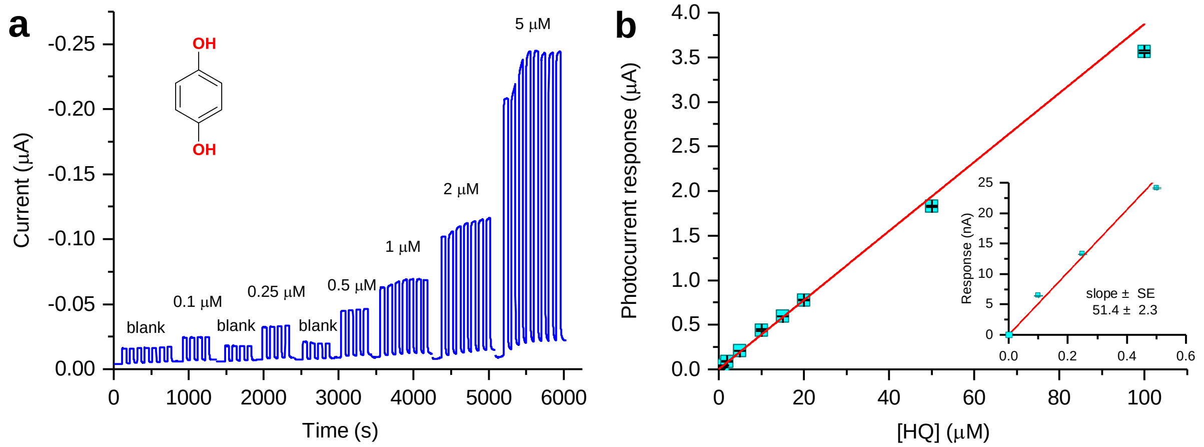

Supplementary Figure 7. Photocurrent response of HQ. Amperometry measurements (**a**) and the calibration curve (**b**) obtained for HQ at −0.1 V in 0.1 M KCl, 0.02 M KH_2PO_4, pH = 7. Sample volume, 80 μL. Diode laser, 655 nm, 30 mW. Data for the calibration curve were presented as mean (±s.d.) of four consecutive measurements.

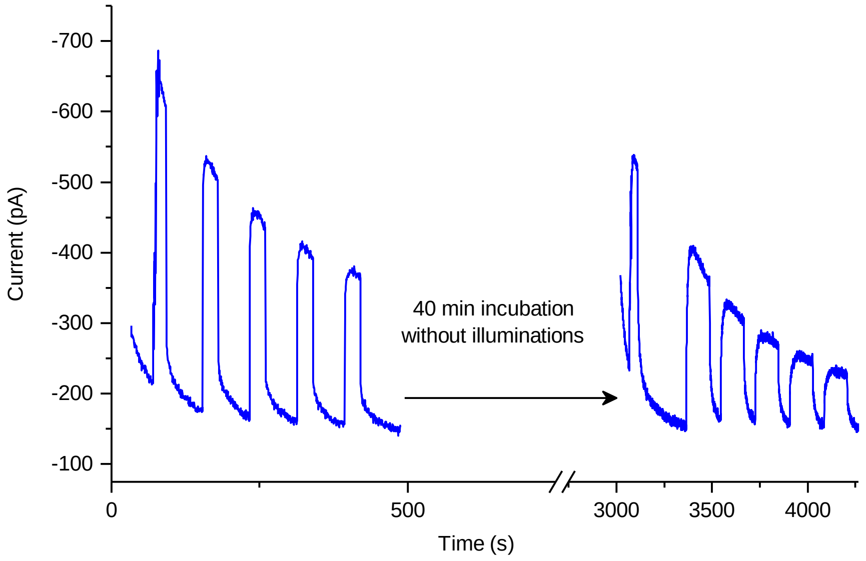

Supplementary Figure 8. Stability of the photosensitizer as a label. The photoelectrochemical response for the oligonucleotide modified electrode after incubation in 10 nM complementary oligonucleotide labeled with Pheophorbide A. Photocurrents were measured in a three-electrode cell at a constant potential of –0.05 V versus SCE using a diode laser (655 nm, 30 mW).

Supplementary Table 1. Photoelectrochemical responses towards phenolic compounds.

	SPE|TiO_2-F_{64}PcZn	SPE|TiO_2-HRP
Hydroquinone	100	100
Catechol	12	38
Phenol	16	35
Bisphenol A	**39**	0.9
2-Chlorophenol	**44**	10
3-Nitrophenol	1.3	0.3
4-Nitrophenol	**13**	0.7
2-Aminophenol	10	26
3-Aminophenol	17	7.5
4-Aminophenol	113	70
2-Amino-4-chlorophenol	19	96
3-Cyanophenol	5.3	No response
4-Cyanophenol	6.5	No response
4-Methylphenol	1.2	0.7
Amoxicillin	**32**	No response
1,1′-Ferrocene-dimethanol	6.3	-
L-Ascorbic acid	No response	-
10x L-Ascorbic acid	1.0	-
100x L-Ascorbic acid	7.4	-
Hydroquinone + L-Ascorbic acid (1:1)	98	-
Hydroquinone + L-Ascorbic acid (1:10)	80	-
Hydroquinone + L-Ascorbic acid (1:100)	51	-
HQ (pH 12)	No response	-
Catechol (pH 12)	No response	-
Phenol (pH 12)	**86**	-
Bisphenol A (pH 12)	**213**	-

Relative responses of SPE|TiO_2-F_{64}PcZn and SPE|TiO_2-HRP modified electrodes to phenolic compounds present in 10 μM concentration. Hydroquinone was assigned a response factor of 100 for both SPE|TiO_2-F_{64}PcZn and SPE|TiO_2-HRP. 1,1'-Ferrocene dimethanol is included for comparison as a one-electron donor while ascorbic acid, which oxidizes irreversibly, is included as a singlet oxygen quencher.

Supplementary Table 2. Performance of HRP-based electrodes designed for detection of phenols.

Method	Electrode/Modification	Sensitivity[a] (nA/μM/cm^2)	Linear range (μM)	Limit of detection (μM)	Ref
Amperometry at -0.1 V "in drop" combined with photocatalytic oxidation.	Screen-printed electrode; commercial TiO_2 was impregnated with the photocatalyst and drop casted on the electrode surface.	410 (HQ)<br>49[b](catechol)<br>66[b](phenol)<br>180[b](2-CP)<br>41[b] (2-AP)	0.1-100 (HQ)	0.012 (HQ)	This work
Amperometry at -0.05 V and continuous stirring at 600 rpm; three-electrode cell.	Modified gold disk electrode; HRP was incorporated in a CNT/polypyrrole nanocomposite matrix.	255 (HQ)<br>64 (catechol)<br>32 (phenol)<br>255 (2-CP)<br>1273 (2-AP)	16-240 (HQ)<br>1.8-8 (catechol)<br>16-44 (phenol)<br>1.6-8 (2-CP)<br>8-60.8 (2-AP)	6.4 (HQ)<br>0.93 (catechol)<br>3.5 (phenol)<br>0.26 (2-CP)<br>1.53 (2-AP)	1
Amperometry at -0.05 V in a mechanically stirred three-electrode cell.	Layer-by-layer self-assembly of HRP multilayers built-up on a thiol-modified gold electrode.	79.6 (HQ)<br>76.4[b](catechol)<br>25.2[b] (phenol)<br>56.1[b] (2-CP)<br>92.2[b] (2-AP)	6-120 (catechol)	0.7 (catechol)	2
Amperometry at 0 V; flow-injection analysis at flow rate of 0.8 ml/min	Modified carbon paste electrode; HRP was immobilized onto SiO_2/Nb_2O_5 sol/gel matrix by adsorption and cross-linking with glutaraldehyde.	235.7[b] (HQ)<br>107.5[b] (catechol)<br>45.3 (phenol)	5-25 (phenol)	0.5 (phenol)	3
Amperometry at -0.05 V in a stirred electrochemical cell.	Modified carbon paste electrode; HRP immobilized onto silica–titanium containing DNA additive and cross-linked with glutaraldehyde.	38.0[b] (catechol)			4
Amperometry at -0.05 V; flow-injection analysis at flow rate of 0.25 ml/min.	HRP adsorbed on a graphite disk electrode.	110.9 (catechol)<br>27.4 (phenol)<br>488 (2-AP)		1.3 (catechol)<br>3.6 (phenol)<br>0.1 (2-AP)	5
Amperometry at -0.05 V in a mechanically stirred electrochemical cell. In situ generation of H_2O_2 by glucose oxidase-catalyzed oxidation of glucose.	Layer-by-layer assembly of concanavalin A and HRP and glucose oxidase on a polyelectrolyte precursor film-modified gold electrode.	50.2 (HQ)<br>120.0 (catechol)<br>23.2 (phenol)<br>76.6 (2-CP)<br>323.3 (2-AP)	6-60 (catechol)	0.9 (catechol)	6
Amperometry at -0.05 V in a three-electrode cell.	Modified carbon paste electrode. MWCNT were chemically modified with methylene blue. HRP was immobilized on the modified MWCNT by cross-linking with glutaraldehyde in the presence of BSA.	50 (catechol)<br>40.3[b] (HQ)<br>18.2[b] (phenol)	1-150 (catechol)	0.5 (catechol)	7
Amperometry at -0.05 V in a mechanically stirred electrochemical cell.	A layer-by-layer self-assembly of concanavalin and HRP on the surface of a thiol-modified gold electrode.	609 (HQ)<br>250 (catechol)<br>81.3 (phenol)<br>594 (2-AP)<br>117.2 (2-CP)	6-72 (HQ)<br>6-48 (catechol)<br>7.6-68.4 (phenol)<br>3.7-25.9 (2-AP)<br>6.9-48.3 (2-CP)	0.2 (HQ)<br>0.6 (catechol)<br>2 (phenol)<br>0.5 (2-AP)<br>2 (2-CP)	8
Amperometry at 0 V; flow-	HRP adsorbed on SWCNT	83.6 (catechol)		0.11 (catechol)	9

injection analysis at flow rate of 0.225 ml/min.	modified screen-printed carbon electrodes.				
Amperometric responses were measured in a stirred cell by applying a potential of −0.05 V.	Co-immobilization of HRP and methylene blue with chitosan on Au-modified TiO_2 nanotube arrays. The titania nanotube arrays were directly grown on a Ti substrate using anodic oxidation first; a gold thin film was then coated onto the TiO_2 nanotubes by an argon plasma technique.	475[10] (phenol)	0.8-130[10] (phenol)	0.2 (phenol)	10
Amperometry at 0 V in a three electrodes cell.	Modified carbon paste electrode; HRP was immobilized on silica gel modified with titanium oxide.	16.3 (phenol)<br>39[b] (HQ)<br>80[b] (catechol)	10-50 (phenol)	1 (phenol)	11

[a]If a sensitivity value was reported in nA/μM, the sensitivity in nA/μM/cm^2 was recalculated from a corresponding electrode surface area. [b]Recalculated values from relative response obtained for different phenolic compounds at a single concentration. Abbreviations: 2-AP – 2-aminophenol; 2-CP – 2-chloroophenol; HQ – hydroquinone; CNT – carbon nanotubes; MWCNT – multi-walled carbon nanotubes SWCNT – single-walled carbon nanotubes.

Supplementary Table 3. Amoxicillin detection. Comparison of the presented approach and other electrochemical methods used for amoxicillin detection.

Method	Electrode/Modification	Linear range (μM)	LOD (μM)	Ref
Photocatalytic oxidation of amoxicillin followed by amperometric detection at -0.1 V "in drop".	Screen printed carbon electrode; TiO_2 impregnated with F_{64}PcZn. The modified TiO_2 was drop-casted on the working electrode.	0.02-100	0.022	This work
SWV; oxidation peak at 0.56 V.	Unmodified edge plane pyrolytic graphite electrode.	5–500	0.84	12
Cathodic accumulation followed by determination at a potential of +0.23V by SWV.	Glutaraldehyde cross-linked polyglutamic acid modified glassy carbon electrode.	2.0–25	0.92	13
SWV measurements of an increase in the peak current for the electroactive complex of oxovanadium (IV) in the presence of amoxicillin.	Carbon paste electrode modified by a complex of oxovanadium(IV).	19–92	8.5	14
Measurement of the oxidation peak of amoxicillin by DPV.	GCE modified by MWCNT decorated with $FeCr_2O_4$ nanoparticles.	0.1–10	0.05	15
Adsorptive stripping CV determination of the oxidation peak of amoxicillin at 0.55 V.	GCE modified by MWCNT.	0.6–8	0.2	16
Measuring the peak current at	Carbon ionic liquid electrode.	5–400	0.8	17

1.08 V by CV.				
Anodic oxidation of amoxicillin in 0.1 M H_2SO_4 by CV.	A poly (N-vinyl imidazole) modified carbon paste electrode.	1–10	0.81	18
Potentiometry in mechanically stirred solution.	Platinum electrode modified by galvanostatic Entrapment of penicillinase into polypyrrole film.	7.5–146	0.3	19
Pre-oxidation of amoxicillin in a MnO_2 reactor followed by amperometric detection using flow-injection analysis.	Unmodified screen-printed carbon electrode combined with a MnO_2 flow reactor.	0.1–100	0.017	20

SWV – square wave voltammetry; DPV – differential pulse voltammetry; CV – cyclic voltammetry; GCE – glassy carbon electrode; MWCNT – multi-walled carbon nanotubes SWCNT – single-walled carbon nanotubes.

Supplementary Methods

Reagents. Oligonucleotides were obtained from Eurogentec (Belgium). Their structures and purity were confirmed by mass-spectrometry. Sequences of the oligonucleotides were as follows: probe 1 (complementary): 5'-HS-$(CH_2)_6$-tagcttatcagactgatgttga-3; probe 2 (non-complementary): 5'-HS-$(CH_2)_6$-tagcttatgtgtaccctgtcag-3'; oligonucleotide labeled by pheophorbide a: 5'-Pheo-tcaacatcagtctga-3'.

Immobilization and hybridization buffers contained 500 mM KCl, 50 mM $MgCl_2$, 10 mM tris pH 7.5. The washing buffer had the same composition, but additionally contained 0.05% w/v tween 20. The measuring buffer contained 0.1 M KCl, 10 mM KH_2PO_4 and had pH = 7.0.

Modification of electrodes. Prior to their modification the gold disks electrodes (1.6 mm in diameter, obtained from BASi, West Lafayette, USA) were polished using diamond paste containing solids of 3, 1 and 0.25 μm particle size dispersed in an alcohol-based lubricant (DP-lubricant Struers, Ballerup, Denmark). Additional polishing was performed with an aqueous 0.05 μm particle size γ-alumina slurry (SPI Supplies, West Chester, PA, USA). Next, the electrodes were placed in 0.5 M H_2SO_4 and subjected to a cyclic potential sweep from 0.2 to 1.45 V versus SCE with a sweep rate of 0.1 V s^{-1} until a steady-state voltammogram was obtained.

For the electrodes modification a thiolated DNA-probe (0.1 mM stock solution in ultrapure water) was mixed in 1:1 ratio with a freshly prepared 10 mM tris(2-carboxyethyl)phosphine (TCEP) in the immobilization buffer. After 1 hour incubation at room temperature, the mixture was diluted 50 times with the immobilization buffer containing 0.5 μM 6-mercaptohexanol (MH). The concentration of the DNA-probe and MH in the final solutions were 1 μM and 0.5 μM, respectively. A 20 μl drop was placed on freshly prepared gold disk electrodes and left overnight at room temperature in a tightly closed chamber that prevented water evaporation. The

electrodes were subsequently washed five times with immobilization buffer and incubated for 2 hours in 1 mM MH immobilization buffer solution.

Detection of labeled oligonucleotides. Electrodes were washed five times with copious amounts of hybridization buffer and placed in the hybridization buffer solution of the labeled oligonucleotide for two hours, followed by immersion in a beaker with washing buffer solution and stirred magnetically at 350 rpm for 5 min. Photocurrents were measured in a three-electrode cell at a constant potential of –0.05 V versus SCE using a red light laser (655 nm, 30 mW). If not used immediately, the electrodes were kept in the measuring buffer for maximum 10 min.

Supplementary References

1. Korkut S, Keskinler B, Erhan E. An amperometric biosensor based on multiwalled carbon nanotube-poly(pyrrole)-horseradish peroxidase nanobiocomposite film for determination of phenol derivatives. *Talanta* **76**, 1147-1152 (2008).

2. Yang SM, Li YM, Jiang XM, Chen ZC, Lin XF. Horseradish peroxidase biosensor based on layer-by-layer technique for the determination of phenolic compounds. *Sens. Actuators, B* **114**, 774-780 (2006).

3. Rosatto SS, Sotomayor PT, Kubota LT, Gushikem Y. SiO2/Nb2O5 sol-gel as a support for HRP immobilization in biosensor preparation for phenol detection. *Electrochim. Acta* **47**, 4451-4458 (2002).

4. Mello LD, Sotomayor M, Kubota LT. HRP-based amperometric biosensor for the polyphenols determination in vegetables extract. *Sens. Actuators, B* **96**, 636-645 (2003).

5. Munteanu FD*, et al.* Bioelectrochemical monitoring of phenols and aromatic amines in flow injection using novel plant peroxidases. *Anal. Chem.* **70**, 2596-2600 (1998).

6. Chen Z, Xi F, Yang S, Wu Q, Lin X. Development of a bienzyme system based on sugar-lectin biospecific interactions for amperometric determination of phenols and aromatic amines. *Sens. Actuators, B* **130**, 900-907 (2008).

7. Santos AS, Pereira AC, Sotomayor MDRT, Tarley CRT, Duran N, Kubota LT. Determination of phenolic compounds based on co-immobilization of methylene blue and HRP on multi-wall carbon nanotubes. *Electroanalysis* **19**, 549-554 (2007).

8. Yang S, Chen Z, Jin X, Lin X. HRP biosensor based on sugar-lectin biospecific interactions for the determination of phenolic compounds. *Electrochim. Acta* **52**, 200-205 (2006).

9. Chekin F, Gorton L, Tapsobea I. Direct and mediated electrochemistry of peroxidase and its electrocatalysis on a variety of screen-printed carbon electrodes: amperometric hydrogen peroxide and phenols biosensor. *Anal. Bioanal. Chem.* **407**, 439-446 (2015).

10. Kafi AKM, Chen A. A novel amperometric biosensor for the detection of nitrophenol. *Talanta* **79**, 97-102 (2009).

11. Rosatto SS, Kubota LT, Neto GD. Biosensor for phenol based on the direct electron transfer blocking of peroxidase immobilising on silica-titanium. *Anal. Chim. Acta* **390**, 65-72 (1999).

12. Rosy, Goyal RN. Estimation of Amoxicillin in Presence of High Concentration of Uric Acid and Other Urinary Metabolites Using an Unmodified Pyrolytic Graphite Sensor. *J. Electrochem. Soc.* **162**, G8-G13 (2015).

13. Santos DP, Bergamini MF, Zanoni MVB. Voltammetric sensor for amoxicillin determination in human urine using polyglutamic acid/glutaraldehyde film. *Sens. Actuators, B* **133**, 398-403 (2008).

14. Bergamini MF, Teixeira MFS, Dockal ER, Bocchi N, Cavalheiro ETG. Evaluation of different voltammetric techniques in the determination of amoxicillin using a carbon paste electrode modified with N,N '-ethylenebis(salicylideneaminato) oxovanadium(IV). *J. Electrochem. Soc.* **153**, E94-E98 (2006).

15. Ensafi AA, Allafchian AR, Rezaei B. Multiwall carbon nanotubes decorated with FeCr2O4, a new selective electrochemical sensor for amoxicillin determination. *J. Nanopart. Res.* **14**, 1244 (2012).

16. Rezaei B, Damiri S. Electrochemistry and Adsorptive Stripping Voltammetric Determination of Amoxicillin on a Multiwalled Carbon Nanotubes Modified Glassy Carbon Electrode. *Electroanalysis* **21**, 1577-1586 (2009).

17. Absalan G, Akhond M, Ershadifar H. Highly sensitive determination and selective immobilization of amoxicillin using carbon ionic liquid electrode. *J. Solid State Electrochem.* **19**, 2491-2499 (2015).

18. Uslu B, Biryol İ. Voltammetric determination of amoxicillin using a poly (N-vinyl imidazole) modified carbon paste electrode. *J. Pharm. Biomed. Anal.* **20**, 591-598 (1999).

19. Ismail F, Adeloju SB, Moline AN. Fabrication of a Single Layer and Bilayer Potentiometric Biosensors for Penicillin by Galvanostatic Entrapment of Penicillinase into Polypyrrole Films. *Electroanalysis* **26**, 2607-2618 (2014).

20. Chiu M-H, Chang J-L, Zen J-M. An Analyte Derivatization Approach for Improved Electrochemical Detection Amoxicillin. *Electroanalysis* **21**, 1562-1567 (2009).
